# Calpains are involved in asexual and sexual development, cell wall integrity and pathogenicity of the rice blast fungus

**DOI:** 10.1038/srep31204

**Published:** 2016-08-09

**Authors:** Xiao-Hong Liu, Guo-Ao Ning, Lu-Yao Huang, Ya-Hui Zhao, Bo Dong, Jian-Ping Lu, Fu-Cheng Lin

**Affiliations:** 1 State Key Laboratory for Rice Biology, Biotechnology Institute, Zhejiang University, Hangzhou 310058, Zhejiang Province, China; 2 State Key Laboratory of Breeding Base for Zhejiang Sustainable Pest and Disease Control, Institute of Virology and Biotechnology, Zhejiang Academy of Agricultural Sciences, Hangzhou 310021, Zhejiang Province, China; 3 College of Life Sciences, Zhejiang University, Hangzhou 310058, Zhejiang Province, China

## Abstract

Calpains are ubiquitous and well-conserved proteins that belong to the calcium-dependent, non-lysosomal cysteine protease family. In this study, 8 putative calpains were identified using Pfam domain analysis and BlastP searches in *M. oryzae*. Three single gene deletion mutants (Δ*Mocapn7*, Δ*Mocapn9* and Δ*Mocapn14*) and two double gene deletion mutants (Δ*Mocapn4*Δ*Mocapn7* and Δ*Mocapn9*Δ*Mocapn7*) were obtained using the high-throughput gene knockout system. The calpain disruption mutants showed defects in colony characteristics, conidiation, sexual reproduction and cell wall integrity. The mycelia of the Δ*Mocapn7*, Δ*Mocapn4*Δ*Mocapn7* and Δ*Mocapn9*Δ*Mocapn7* mutants showed reduced pathogenicity on rice and barley.

In eukaryotic cells, there are several protein degradation systems, including autophagy, the ubiquitin-proteasome system (UPS), cytosolic proteases, such as calpains and caspases, and organelle-specific degradation systems. These degradation systems cooperate and function simultaneously. For decades, studies have focused on analyzing the mechanisms and functions of the two major protein degradation processes, autophagy and the UPS, in eukaryotic cells. Disruption of these processes results in dysfunctional cellular homeostasis and energy balance^1^,^2^,^3^,^4^,^5^,^6^,^7^. However, another major factor in protein degradation, the calpain system, has been overlooked. The physiological role of calpains is still poorly understood in eukaryotic cells^8^,^9^.

Calpains are ubiquitous and well-conserved proteins and belong to the calcium-dependent, non-lysosomal cysteine protease family^8^. In pioneering studies initiated in 1964, calpains were detected in human tissues. Calpains are involved in physiological and pathophysiological processes, such as cytoskeletal reorganization, signal transduction pathways, cell cycle regulation and specific apoptosis pathways. Calpains have been shown to play important roles in human pathogenesis, and calpain dysfunction is related to specific diseases, such as cataracts, Parkinson’s disease and Alzheimer’s disease^10^.

Thus far, calpain homologs have been identified in almost all eukaryotes and a few bacteria. Approximately 15 calpain proteins have been found in humans; 9 of them are considered classical [contain CBSW (calpain-type beta-sandwich domain), PEF (penta-EF-hand domain) and CysPc (calcium-dependent cytoplasmic cysteine proteinases) domain], and 6 are non-classical (do not contain CBSW and PEF). Most of the classical human calpains are conserved in other vertebrates^11^,^12^. However, only a few classical calpains have been identified in other organisms, and no classical calpain homologs have been reported in fungi. In *Saccharomyces cerevisiae*, only one putative gene (designated CPL1) was identified as a cysteine protease with a protease domain similar to that of calpain^13^. Cpl1p shows significant sequence similarity to PalBp, a fungal (*Aspergillus nidulans*) calpain-like protease that is responsible for adaptation under alkaline conditions, both in the protease domain and the domain following the protease domain^14^. In addition, this gene product is homologous to human Capn7 (PalBH), the most evolutionarily conserved human calpain, which has been found in vertebrates, fungi, protists, nematodes, and insects^15^,^16^.

*Magnaporthe oryzae* is considered an excellent model for studying the interactions between plants and fungi^17^. It infects many cereal crops, such as rice and barley, and causes rice blast disease which is the most economically devastating disease of cultivated rice. The rice blast fungus infects its host through a specialized infection structure called appressorium that attaches tightly to the plant surface and directs the penetration into the host tissue by breaching the cuticle and the plant cell wall. Reorganization, signal transduction and material turnover are involved in the typical infectious cycle of *M. oryzae*^18^,^19^. As previously reported, protein degradation pathways, such as the UPS and autophagy, play pivotal roles in the nutrient assimilation, development and pathogenicity of *M. oryzae*^2^,^20^,^21^,^22^,^23^,^24^. As described in our previous report, the homologs of human calpains 1, 3 and 4, designated *MoCAPN1, MoCAPN3 and MoCAPN4*, respectively, have been studied in *M. oryzae*. Following disruption of the calpain genes, conidiation and pathogenicity were impaired^25^.

In this study, we focused on the calpains, another type of protein degradation factor in *M. oryzae*. Eight putative calpains were identified in the *M. oryzae* proteome using BlastP. Additionally, we obtained 3 single gene deletion mutants and 2 double gene deletion mutants using the high-throughput gene knockout system. Further analysis showed that calpains play multiple roles in conidiation, sexual reproduction, cell wall integrity and pathogenicity.

## Results

### Identification and expression profiles of the calpains in *M. oryzae*

Pfam domain analysis and BlastP searches were used to identify the calpain genes in the Broad Institute *M. oryzae* protein database (http://www.broad.mit.edu). Pfam domain analysis of the *M. oryzae* proteome was performed using the integrated module of Pfam domain search in the CLC Genomics Workbench (QIAGEN) with default parameters. BlastP analysis is used to establish sequence relationships, with an e-value of 0.001 or lower defined as statistically significant. Using the sequences of the 15 human calpains against the *M. oryzae* genome, we identified 8 putative calpains as shown in Table S1. Classical calpains contain CysPc domains (CysPc, calpain-like cysteine protease sequence motif) and PEF domain (penta-EF-hand, calcium-binding motif). Among these putative calpains in *M. oryzae*, MGG_15810, MGG_14872, MGG_07573, MGG_06335 and MGG_08526 contain the CysPc domain and the peptidase_C2 domain. The EF-hand domains were found only in MGG_04818, MGG_01072 and MGG_16201 (Table S1). Thus, all putative calpain proteins identified in *M. oryzae* belong to the non-classical calpains. The functions of proteins MGG_15810, MGG_14872 and MGG_04818, the homologs of human calpains 1, 3 and 4, have been studied previously^25^. Based on the sequence identies and similarities, MGG_08526, MGG_06335, MGG_01072, and MGG_07573 are homologues of human calpains 2, 7, 9, and 14, respectively (Table S1). MGG_16201 has similarity to calpain 1 and 3 and was therefore named Mocapn1B (Table S1).

**Figure 1:**
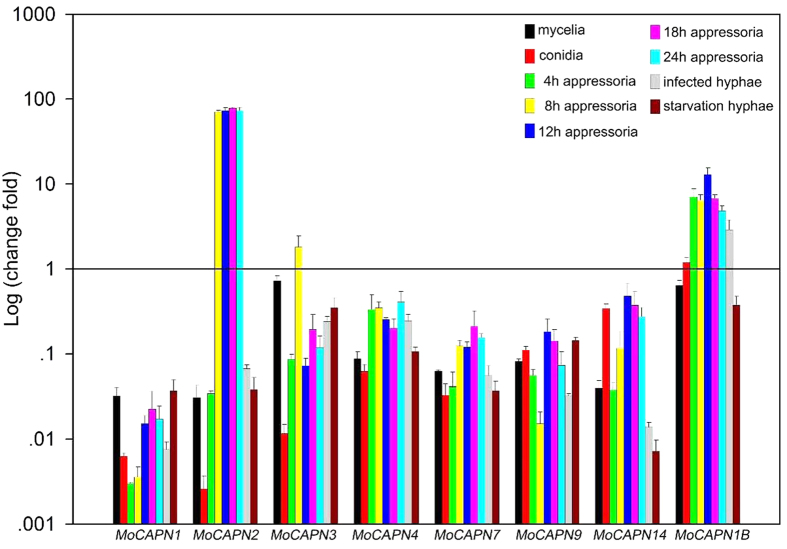
The expression profiles of the *M. oryzae* calpains in development, pathogenicity and starvation stress. qRT-PCR assays were carried out with RNA samples obtained from different stages of the wild-type strain, including vegetative hyphae, conidia, appressoria, infected hyphae and nitrogen-starvation hyphae. Gene expression levels were normalized using the β-tubulin gene as an internal standard. Data are representative of at least two independent experiments with similar results, and the error bars represent the standard deviations of three replicates.

To determine the expression profiles of the *M. oryzae* calpains during development (vegetative hyphae, conidia, appressoria), pathogenicity (infected hyphae) and starvation stress (nitrogen-starvation hyphae), calpain genes expressions were evaluated using qRT-PCR assays (Fig. 1). The expression levels of the *MoCAPN1, 3, 4, 7* and *9* genes showed very low expression during development, pathogenicity and starvation stress (down-regulation more than 10-fold). In contrast, the *MoCAPN2* gene displayed very high expression levels (up-regulation more than 70-fold) in appressoria from the initial appressoria (4 h) to the mature appressoria (24 h). In addition, the *MoCAPN1B* gene was highly expressed in appressoria and infected hyphae, especially in the mid-development appressoria (up-regulation more than 10-fold). As a result, the calpain genes were shown to have diverse gene expression patterns in *M. oryzae. MoCAPN2* and *MoCAPN1B* had high expression levels in appressoria.

### Deletion of the calpain genes in *M. oryzae*

**Figure 2:**
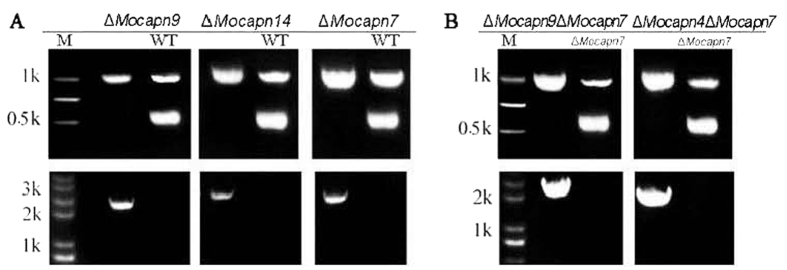
Knockout event of null mutants confirmed by PCR. (**A**) The single-gene-deletion mutants, Δ*Mocapn7*, Δ*Mocapn9*, Δ*Mocapn14*. (**B**) The double-gene-deletion mutants, Δ*Mocapn4*Δ*Mocapn7* and Δ*Mocapn9*Δ*Mocapn7*. The mutants were verified by double PCR for the targeted gene using the β-tubulin gene as a positive control (~1.0 kb band). The wild-type strain produced a characteristic band, indicating the targeted gene (~0.5 kb band), while the null mutants did not (Upper). The mutants were verified by PCR using a unique recombinational DNA fragment indicating a knockout event. The null mutants have a ~2.0–3.0 kb band on an electrophoretic gel, while the wild-type strain and the ectopic transformants do not (Lower).

To confirm the functions of the calpains in the development and pathogenicity of the rice blast fungus, we deleted the calpain genes in *M. oryzae* using the high-throughput gene knockout system via the pKO1B vector as described in the Experimental procedures. The deletion vectors MGG_06335-HPH-pKO1B, MGG_01072-HPH-pKO1B, MGG_07573-HPH-pKO1B, MGG_08526-HPH-pKO1B and MGG_16201-HPH-pKO1B were transformed into the Guy11 conidia using the ATMT method. Hygromycin-resistant transformants grown on CM medium were initially screened under a fluorescence microscope. The transformants without green fluorescence were analyzed for the target gene and *β-tubulin* gene (control) using a double PCR protocol. The wild-type strain or ectopic transformants produced a characteristic band, indicating the targeted gene, while the null mutants did not (Fig. 2A). Then, the candidate null mutants were screened by PCR for a unique recombinant DNA fragment indicating a knockout event. The true null mutants had a 2.0–3.0 kb band on gel electrophoresis, while the wild-type strain and the ectopic transformants did not (Fig. 2A). qPCR analysis confirmed that the null mutants contained a single copy of a gene knockout cassette that was integrated into the genomic DNA (data no shown). Southern blot assays were performed to re-confirm the single copy genomic integration and exclude additional ectopic integrations (Figure S1). Finally, the mutants containing a single copy of the gene deletion cassette were considered as the null mutants (Δ*Mocapn7*, Δ*Mocapn9*, Δ*Mocapn14*). Unfortunately, we did not obtain the null mutant of the genes *MoCAPN2 (MGG_08526*) and *MoCAPN1B (MGG_16201*).

To confirm the synergistic effects of the CysPc and EFs domains in *M. oryzae*, double gene knock-out vectors (MGG_01072-BAR-pKO1B and MGG_04818-BAR-pKO1B) were generated. The vectors were transformed into the conidia of the Δ*Mocapn7* mutant using the ATMT method. Glufosinate ammonium-resistant transformants were screened and confirmed using the same methods described above. Then the double-gene knock-out mutants, Δ*Mocapn4*Δ*Mocapn7* and Δ*Mocapn9*Δ*Mocapn7* were generated (Fig. 2B). All the mutants were then purified by mono-conidial isolation and further analyzed for phenotypic studies.

### Calpains have multiple effects on colony characteristics and conidiation

**Figure 3:**
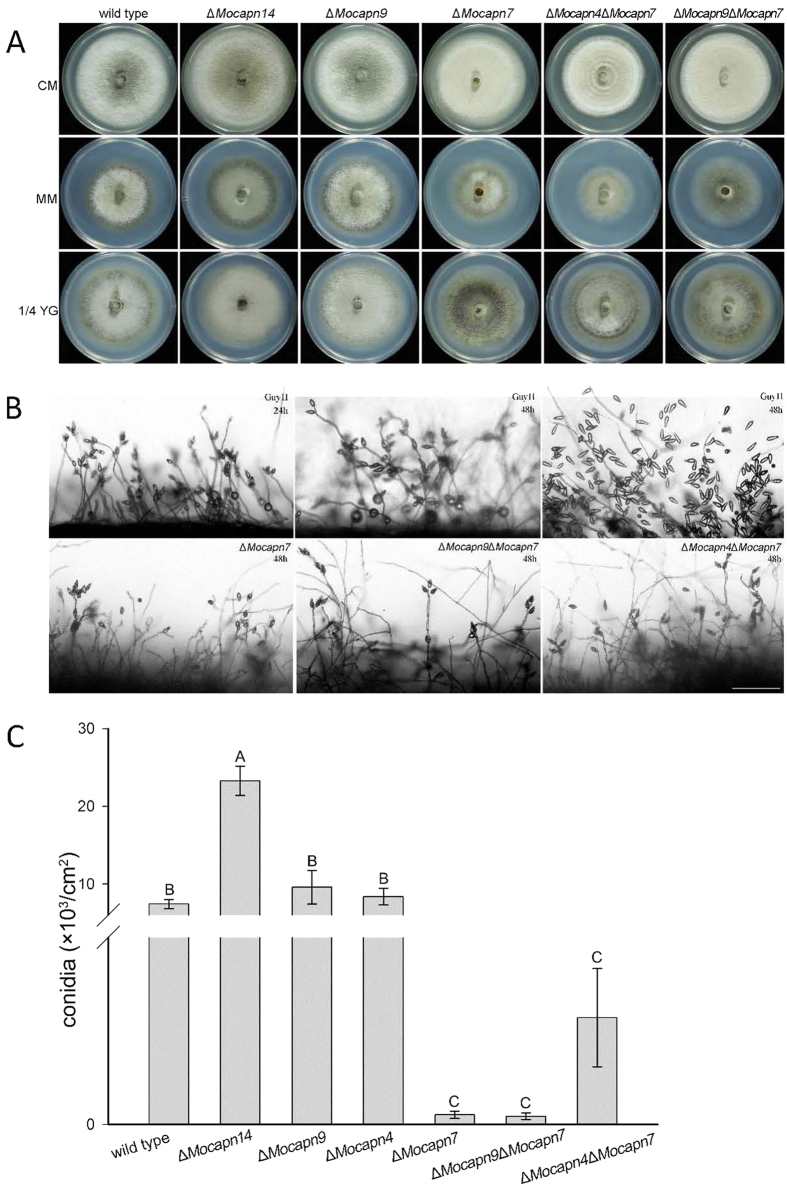
(**A**) Characteristics of the strains. The strains were grown on CM, MM, and 1/4 YG medium for 7 days. (**B**) Development of conidia on conidiophores observed under a light microscope under cover slips 24 h or 48 h after induction of conidiation. A few conidia developed in the Δ*Mocapn7*, Δ*Mocapn9*Δ*Mocapn7* and Δ*Mocapn4*Δ*Mocapn7* mutants. Scale bar = 100 μm. (**C**) The production of conidiation. Error bars represent the standard deviation (P < 0.01). Different letters indicate a statistically significant difference.

The phenotypes of the calpain gene deletion mutants were observed. The colonies of the Δ*Mocapn9* and Δ*Mocapn14* mutants were similar to the colonies of the wild-type strain Guy11 on CM, OMA and 1/4 YG media. However, the effects of *MoCAPN7* gene deletion on morphology and development were dramatic. The Δ*Mocapn7*, Δ*Mocapn4*Δ*Mocapn7* and Δ*Mocapn9*Δ*Mocapn7* mutants had grey-white aerial hyphae, which contrasted with the dark aerial hyphae of the wild-type strain Guy11. Interestingly, the aerial hyphae of Δ*Mocapn4*Δ*Mocapn7* were denser than those of Δ*Mocapn7* or Δ*Mocapn9*Δ*Mocapn7* on different media (Fig. 3A).

Conidiophores and conidial formation of the mutants were subsequently analyzed. The Δ*Mocapn9* and Δ*Mocapn14* mutants were similar with the wild-type strain Guy11 and developed multiple conidiophores with pyriform conidia sympodially arrayed at 24 h post-conidial induction (Fig. 3B). However, the Δ*Mocapn7*, Δ*Mocapn4*Δ*Mocapn7* and Δ*Mocapn9*Δ*Mocapn7* mutants had few and short aerial hyphae at 24 h post-conidial induction. After prolonged incubation for 48 h, the length of the aerial hyphae increased significantly and produced a few conidia (Fig. 3B). The Δ*Mocapn9* mutant produced 3 times more conidia than Guy11. However, the Δ*Mocapn7* mutant produced 1/70 as many conidia as Guy11. Similarly, Δ*Mocapn4*Δ*Mocapn7* and Δ*Mocapn9*Δ*Mocapn7* mutants produced fewer conidia. In contrast to Δ*Mocapn7*, Δ*Mocapn4*Δ*Mocapn7* produced 10 times more conidia than Δ*Mocapn7.* The conidia production increased following deletion of the *MoCAPN4* gene in the Δ*Mocapn7* mutant (Fig. 3C). These results suggested that the calpains play pleiotropic roles in the development of the rice blast fungus.

### Calpains are important for sexual reproduction

**Figure 4:**
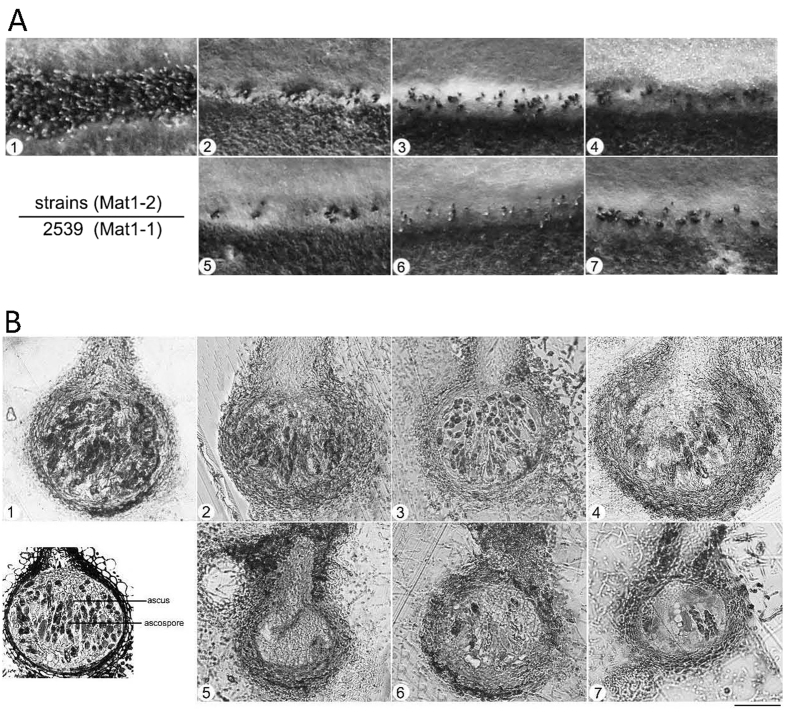
(**A**) Sexual reproduction experiments. Guy11 × 2539 formed numerous perithecia at the junctions between mated individuals on oatmeal medium; a few perithecia were observed for the crosses of Δ*Mocapn14*, Δ*Mocapn7*, Δ*Mocapn9*, Δ*Mocapn9*Δ*Mocapn7* and Δ*Mocapn4*Δ*Mocapn7* × 2539. Arrows indicate perithecia. (**B**) The semithin sections of perithecia. 1, Δ*Mocapn14*; 2, Δ*Mocapn7*; 3, Δ*Mocapn9*; 4, Δ*Mocapn4*; 5, Δ*Mocapn7*; 6, Δ*Mocapn4*Δ*Mocapn7*; 7, Δ*Mocapn9*Δ*Mocapn7*. The diagram of the perithecium is shown on the lower left. Scale bar = 50 μm.

The wild-type strain Guy11 is heterothallic and requires an opposite mating type strain (2539 was used in our study) for sexual reproduction. In sexual reproduction assays, we crossed Guy11 and the calpain gene deletion mutants with the 2539 strain (described in the Experimental procedures). After 4-week incubation, hybridization between Guy11 and 2539 produced abundant and viable melanized perithecia at the borders (Fig. 4A). In contrast, few perithecia were observed in the borders between the calpain-gene-deletion mutants (Δ*Mocapn4*, Δ*Mocapn7*, Δ*Mocapn9*, Δ*Mocapn14*, Δ*Mocapn4*Δ*Mocapn7*, and Δ*Mocapn9*Δ*Mocapn7*) × 2539 (Fig. 4A).

To further explore the functions of the calpains in sexual production, asci and ascospores were examined using semi-thin sections. After 4-week incubation, many mature asci and ascospores developed inside the perithecia of Guy11 × 2539 (Fig. 4B1). However, no normal asci and ascospores could be observed inside the perithecia of calpain gene deletion mutants × 2539. After 6-week incubation, the number of perithecia did not increase in the calpain gene deletion mutants × 2539. Many normal asci and ascospores could be observed inside the perithecia of Δ*Mocapn4* × 2539, Δ*Mocapn9* × 2539, and Δ*Mocapn14* × 2539 (Fig. 4B). In addition, a few asci and ascospores developed from the perithecia of Δ*Mocapn4*Δ*Mocapn7* × 2539 and Δ*Mocapn9*Δ*Mocapn7* × 2539. However, the perithecia of Δ*Mocapn7* × 2539 could not develop asci and ascospores (Fig. 4B). Thus, calpains are required for normal sexual development in *M. oryzae*.

### Deletion of the calpain genes results in cell wall defects

**Figure 5:**
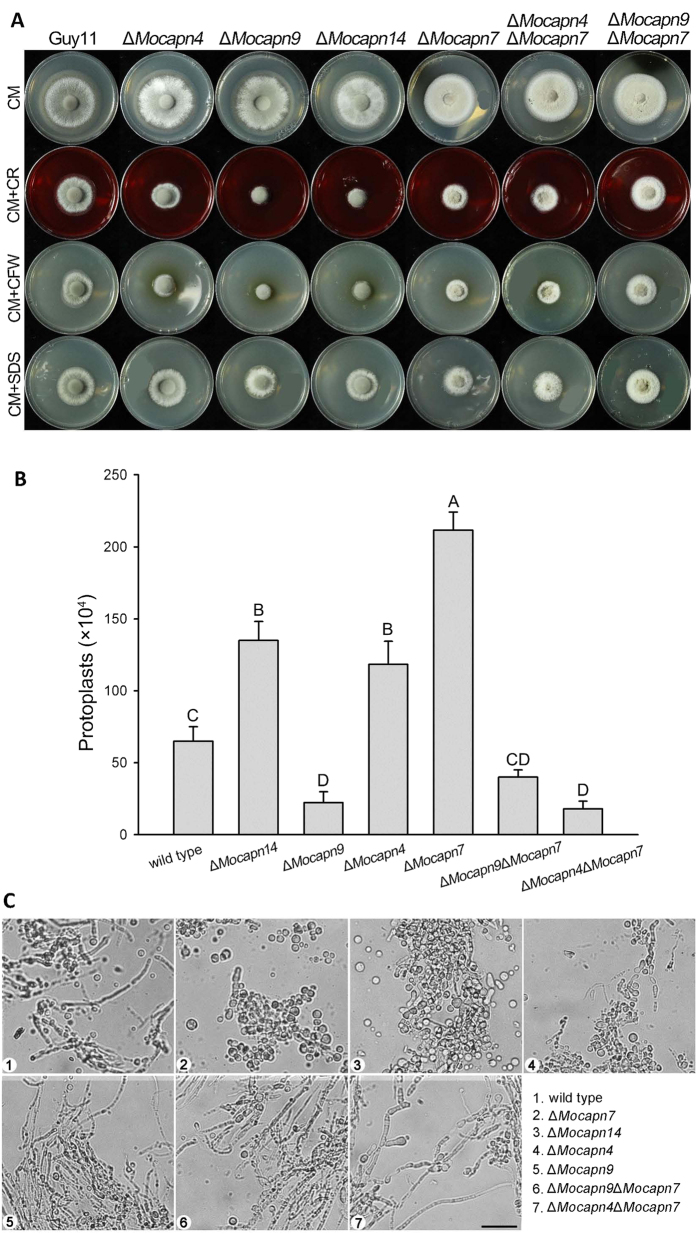
Deletion of calpains resulted in alterations in cell wall integrity. (**A**) *M. oryzae* strains were incubated on CM plates supplemented with various stress inducers at 28 °C for 6 days. Growth of strains in media supplemented with SDS (0.1%), Congo red (200 mg/ml) and CFW (100 μg/ml). (**B**) Sensitivity of *M. oryzae* strains to cell-wall-digesting enzymes. Bars represent the mean number of protoplasts (×10^4^/ml) released by the hyphae of mutants or Guy11 after a 30 min incubation with Glucanex solution. Error bars represent one standard deviation (P < 0.01). Different letters indicate a statistically significant difference. (**C**) Sensitivity of strains to cell-wall-digesting enzymes. The wild-type strain Guy11 and the mutants were photographed after treatment with Glucanex for 30 min. A large number of protoplasts were released from the mycelia of the Δ*Mocapn7*, Δ*Mocapn9*, and Δ*Mocapn14* mutants. Few protoplasts were released from the mycelia of the Δ*Mocapn9*Δ*Mocapn7* and Δ*Mocapn4*Δ*Mocapn7* mutants. Scale bar = 25 μm.

To examine the roles of the calpain genes in cell wall integrity, we assessed the effects of various cell wall perturbing agents on the calpain deletion mutants. Mycelial growth was measured on CM plates amended with calcofluor white (CFW), Congo red and SDS, compounds known to cause cell wall stress^26^. The sensitivity of the mutants (Δ*Mocapn7*, Δ*Mocapn9*, Δ*Mocapn14*) to CFW, SDS and Congo red was significantly different from that of the wild-type strain Guy11 (Fig. 5A). The sensitivity of the double gene deletion mutants (Δ*Mocapn4*Δ*Mocapn7* and Δ*Mocapn9*Δ*Mocapn7*) to CFW was significantly different from that of the wild-type strain Guy11. However, the sensitivity of the Δ*Mocapn4*Δ*Mocapn7* and Δ*Mocapn9*Δ*Mocapn7* mutants to SDS and Congo red was not significantly different from that of the wild-type strain Guy11 (Fig. 5A and Table S6). Thus, the calpain genes have different roles in cell wall integrity.

To further verify the changes in cell wall integrity resulting from the deletion of the calpain genes, cell wall-degrading enzymes (Glucanex, Sigma) were applied to examine the release of protoplasts. Significant differences in the rate of protoplast release were found at 30 min in the Guy11 and mutants (Fig. 5B). The Δ*Mocapn4*, Δ*Mocapn7*, and Δ*Mocapn14* hyphae released more protoplasts than did the wild-type Guy11 (P < 0.01) after 30 min of incubation with a solution of the fungal cell wall degrading enzyme. However, the numbers of the protoplasts produced by the Δ*Mocapn4*Δ*Mocapn7* and Δ*Mocapn9*Δ*Mocapn7* mutants were lower than that of Guy11 and the single gene deletion mutants (Δ*Mocapn4*, Δ*Mocapn7*, and Δ*Mocapn14*) (Fig. 5B). In addition, we observed the morphology of the digest mycelia. The hyphae of Δ*Mocapn4*, Δ*Mocapn7*, and Δ*Mocapn14* fragmented, in contrast to the mostly intact hyphae of Guy11, Δ*Mocapn4*Δ*Mocapn7* and Δ*Mocapn9*Δ*Mocapn7* (Fig. 5C). All these data indicated that the calpain genes deeply influenced on the sensitivity to the cell wall degrading enzymes.

### The pathogenicity of the mutants towards the hosts is impaired

**Figure 6:**
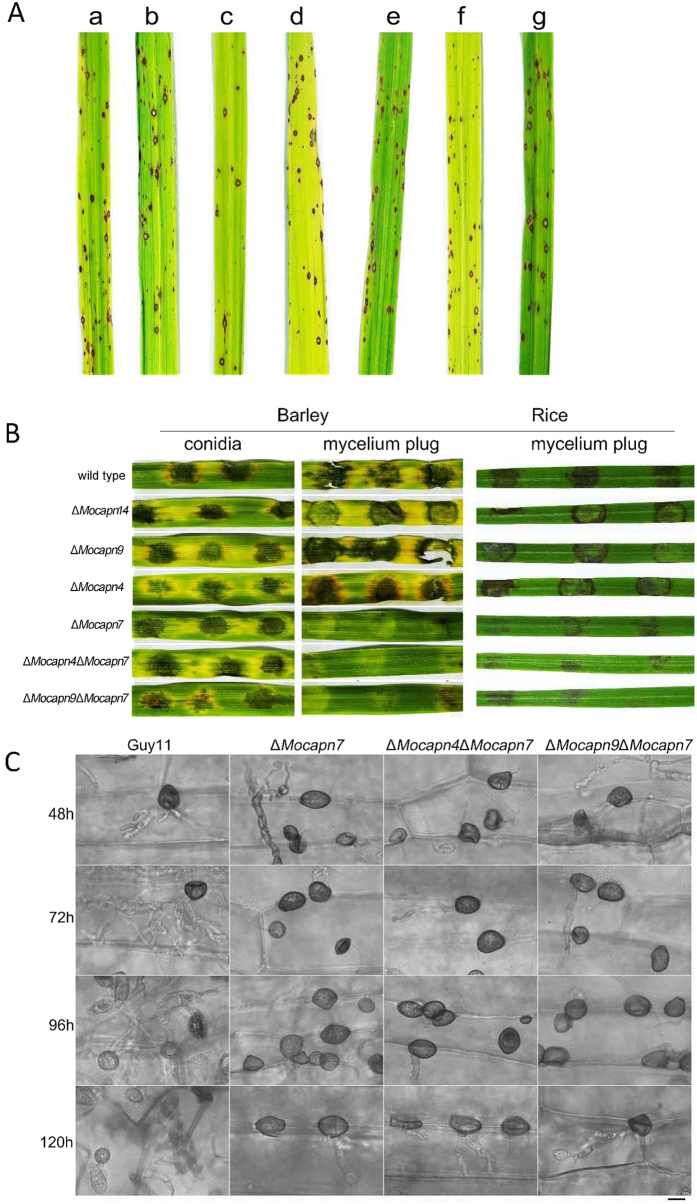
(**A**) Disease symptoms on rice inoculated with conidial suspension from Guy11 and mutants. Typical leaves were photographed 7 days after inoculation. (**B**) Disease symptoms on the cut leaves of barley and rice inoculated with mycelial plugs from Guy11 and mutants. Typical leaves were photographed 4 days after inoculation. (**C**) Development of appressorium-like structures and penetration assays on barley with Guy11 and mutants. Scale bar = 10 μm.

Pathogenicity assays were performed on two different susceptible plant hosts (rice and barley). Very few conidia were produced in the Δ*Mocapn7*, Δ*Mocapn4*Δ*Mocapn7* and Δ*Mocapn9*Δ*Mocapn7* mutants. We obtained 1 × 10^4^ conidia/ml suspensions from the Δ*Mocapn7*, Δ*Mocapn4*Δ*Mocapn7* and Δ*Mocapn9*Δ*Mocapn7* mutants. Following inoculation with the 1 × 10^4^ conidia/ml suspensions of Guy11 and mutants on 14-day-old rice, spindle-shaped lesions were observed on the rice (Fig. 6A). In addition, the 1 × 10^4^ conidia/ml suspensions of the strains were inoculated on the detached leaves of barley. No differences in symptoms were observed on the barley (Fig. 6B, barley). It is suggested that there are no significant differences in appressoria-mediated pathogenicity of calpains.

Mycelial plugs of the strains were inoculated on detached barley leaves. Negligible lesions were produced when the plugs of the Δ*Mocapn7*, Δ*Mocapn4*Δ*Mocapn7* and Δ*Mocapn9*Δ*Mocapn7* mutants were inoculated on the barley leaves, while typical lesions were produced when the plugs of the wild-type strain Guy11, Δ*Mocapn9* and Δ*Mocapn14* were inoculated (Fig. 6B, barley). Similarly, mycelial plugs of the strains were inoculated on the detached leaves of rice. Reduced lesions were produced when the plugs from the Δ*Mocapn7*, Δ*Mocapn4*Δ*Mocapn7* and Δ*Mocapn9*Δ*Mocapn7* mutants were inoculated on detached rice leaves. However, typical lesions were produced when the mycelial plugs of the wild-type strain Guy11, Δ*Mocapn9* and Δ*Mocapn14* were inoculated (Fig. 6B, rice).

To gain further insights into the differences of the disease symptoms on the leaves, the appressorium-like structures (ALS) formed by the hyphal tips were monitored by inoculating barley with mycelial plugs. Interestingly, appressorium-like structures formed by the hyphal tips showed significant differences among the wild-type strain Guy11, Δ*Mocapn7*, Δ*Mocapn4*Δ*Mocapn7* and Δ*Mocapn9*Δ*Mocapn7* (Fig. 6C). After 48 h of inoculation, the hyphal tips of Guy11 developed many appressorium-like structures, which formed typical invasive hyphae in the plant cells. In contrast, typical invasive hyphae were not formed by the appressorium-like structures developed from the hyphal tips of the mutants (Δ*Mocapn7*, Δ*Mocapn4*Δ*Mocapn7* and Δ*Mocapn9*Δ*Mocapn7*). After inoculation for 72 h and 96 h, infectious hyphae formed by Guy11 appressorium-like structures extended between cells. After inoculation for 120 h, new appressorium-like structures were produced by the infectious hyphal tips of Guy11. However, after inoculation for 120 h, few appressorium-like structures were formed by the hyphal tips of the mutants (Δ*Mocapn7*, Δ*Mocapn4*Δ*Mocapn7* and Δ*Mocapn9*Δ*Mocapn7*) (Fig. 6C). It can be inferred that *MoCAPN7* had more severe effects on the development and functions of the appressorium-like structures. Disruption of *MoCAPN7* delayed the development of appressorium-like structures and reduced penetration efficiency.

### Reintroduction of calpains restores the defects of the mutants

Complementation assays were performed to determine whether the observed phenotypes were attributable to calpains disruption. Complementation analysis was performed by introducing a cDNA fragment of each calpain gene into the corresponding null mutant. Transformants were selected, and the presence of a single copy of the calpain gene was confirmed by qPCR. Detached leaves of barley inoculated with mycelial plugs from rescued transformants exhibited normal rice blast disease symptoms. The colony characteristics, conidiation and cell wall integrity were all restored by complementation assays.

## Discussion

The two major protein degradation pathways, autophagy and the UPS in *M. oryzae*, have been intensively studied in the past decades. Autophagy-deficient fungal mutants showed defects in conidiation, turgor pressure and pathogenicity^2^,^21^,^27^,^28^,^29^,^30^. And ubiquitin-mediated proteolysis of target proteins plays an important role in nutrient assimilation, development and pathogenicity of *M. oryzae*^22^,^23^,^31^. In this study, we elucidate the pivotal functions of calpains, an overlooked protein degradation system in *M. oryzae*. The domain organization in putative calpain proteins is schematically represented in *M. oryzae*. The null mutants of the calpain genes were obtained using the high-throughput gene knockout system. Subsequently, we determined that calpains play multiple roles in conidiation, sexual reproduction, cell wall integrity and pathogenicity in *M. oryzae*.

Calpain homologs have been identified in almost all eukaryotes and a few bacteria, but classical calpains possessing PEF domain are not found in fungi. In human, classical calpains contain CBSW, PEF and CysPc domains and non-classical calpains do not contain the CBSW and PEF domains. We found 8 non-classical calpains in *M. oryzae* using Pfam domain analysis and BlastP searches. The identified calpains in *M. oryzae* only contain the CysPc or EFh domain. The fungal orthologs of mammalian calpain-7 (a non-classical calpain), Cpl1 in *S. cerevisiae*, PalB in *A. nidulans* and MoCapn7 in *M. oryzae* lack the PEF domain, but they show high similarity to evolutionarily conserved mammalian calpain-7.

Conidia formation is an essential stage of the infectious cycle in *M. oryzae*. Conidia developed from the expansion and swelling of the apex of the conidiophore. In our analysis of the conidia production, the wild-type strain Guy11 developed many conidiophores with pyriform conidia sympodially arrayed at 24 hpi. However, the Δ*Mocapn7*, Δ*Mocapn4*Δ*Mocapn7* and Δ*Mocapn9*Δ*Mocapn7* mutants formed rare and very short aerial hyphae at 24 hpi. Long aerial hyphae and conidiophores were produced by the mycelia at the colony margin at 48 h hpi, and few conidia was differentiated from conidiophore of the mutants. We deduced that reduced conidiation in the Δ*Mocapn7* resulted from a defect in conidiophore development and differentiation. Consistent with the previous reports, Δ*Mocapn1* mutants showed significantly reduced conidia production compared to that of the wild-type Guy11^25^. Note that the yeast *CPL1* disruption mutant had sporulation deficiency^14^. While, the Δ*Mocapn9* and Δ*Mocapn14* mutants had more conidia than the wild-type Guy11. These findings suggested that the different components of the calpain system have pleiotropic and interacting effects on conidia development of the rice blast fungi. Consistent with the results observed for Δ*Mocapn1* and Δ*Mocapn7*, conidia production was significantly affected in the autophagy-disruption mutants, such as *MoATG1-9, MoATG12, 14, 16* and *18*^2^. In the UPS pathway, MGG_01282, is predicted to encode a polyubiquitin protein, deletion of which results in significant reduction in conidiation. Knockdown of MoSkp1, a core component of the SCF E3 ubiquitin ligase complex, resulted in defects in growth, sporulation and appressorial development^23^. MoGrr1 is one of the F-box proteins which are specific adaptors to E3 ubiquitin ligases. Targeted disruption of *MoGRR1* genes caused defects in conidiophore formation and conidiation^25^. Therefore, various types of protein degradation systems play essential roles in conidia formation in *M. oryzae*.

Sexual reproduction is an ability of organisms to adapt to changing environmental conditions, such as pathogens encountering new hosts or new agricultural practices^32^,^33^. *M. oryzae* is an extremely effective plant pathogen and it can reproduce both sexually and asexually^34^. Our results showed that calpains were closely related to sexual reproduction. Formation of the perithecia was delayed in the calpain deletion mutants. Especially, the Δ*Mocapn7* mutant showed significant defects in formation of the perithecia and ascospores. As our previous studies, formation of the perithecia and ascospores was delayed and weakened in the autophagy blocked mutants, Δ*Moatg1* and Δ*Moatg5*^28^,^30^. Similarly, the Δ*Mogrr1* mutant showed a greater reduction in perithecia^24^, but the ascospores formation was not affected. These findings suggest that the calpains system, like autophagy and UPS, play important roles in sexual reproduction of *M. oryzae*.

The fungal cell wall is the outermost layer exposed to the surrounding environments and is essential for maintaining the cellular structure and integrity of the fungi. It is a matrix with three major components, chitin, glucans and proteins, and represents the first line of defense. Many stresses and developmental processes, as well as plant infection, can induce remodeling of cell walls^35^. In the present study, calpain single gene deletion mutants showed repressed mycelial growth in the presence of CFW and Congo red. Further analysis with cell-wall-digesting assay indicated that those mutants were sensitive to glucannex. However, qRT-PCR analysis revealed no significant difference between the wild-type Guy11 and the calpain mutants in the expression of genes related to cell wall synthesis, such as chitin synthases (data not shown). Therefore, we deduce that calpains may be involved in the regulation of other components in the cell wall. *MPS1* and *MCK1*, the components of MAP signal pathway, required for maintenance of cell wall integrity of *M. oryzae.* Previous studies have shown that Δ*mps1* and Δ*mck1* were hypersensitivity to the cell-wall-digesting enzymes, as found for the calpain knockout mutants. Expression of cell wall synthesis-related genes was also not significantly changed in the Δ*mck1* mutant. Additionally, Δ*mps1* and Δ*mck1* had obvious autolysis phenotype at the center of the mycelium and radiated outward^36^,^37^. Similarly, autolysis phenotype was observed for Δ*Mocapn7* after incubation on CM plates for more than 12 days (data not shown). The similar behaviors of the calpain knockout mutants and Δ*mps1*/Δ*mck1* indicated that the regulation of MAP signal pathway in cell wall integrity was likely impaired in the calpain disruption mutants. Further analysis is necessary to verify the interaction between the MAP signal pathway and calpains in *M. oryzae*.

Interestingly, mycelial growth of the calpain double gene deletion mutants (Δ*Mocapn4*Δ*Mocapn7* and Δ*Mocapn9*Δ*Mocapn7*) was similar to that of the wild-type Guy11 in the presence of CFW and Congo red. And the sensitivity to cell-wall-digesting enzyme was recovered in the double gene deletion mutants (Δ*Mocapn4*Δ*Mocapn7* and Δ*Mocapn9*Δ*Mocapn7*). In addition, conidia increased in the Δ*Mocapn7* mutant following deletion of the *MGG_04818 (MoCAPN4*) gene, although it has been reported that the *MGG_04818 (MoCAPN4*) gene has no effect on conidiogenesis^25^. Conidiophores of the Δ*Mocapn4*Δ*Mocapn7* mutant differentiated more conidia than the Δ*Mocapn7* mutant. A cascade of signal transduction pathway is involved in conidiogenesis and cell wall integrity, such as MAP signal pathway, Ca^2+^ signaling. We deduced that different calpain gene play diverse roles in regulating the signal pathway in *M. oryzae*. It has been reported that homozygous disruption of murine Capn4 eliminated both μ- and m-calpain activities^38^,^39^. Capn4 has been identified as the small regulatory subunit calpain in the proteolytic system of mammals^39^. The *MoCAPN4* gene may be a regulator of the calpain system. Further studies are needed to explore the regulatory functions of *MoCAPN4* or *MoCAPN9*, and the mechanism of calpain regulating the signal pathway in *M. oryzae*.

It has been reported that appressorium formation and the development of appressorium-like structures are not identical processes in *M. oryzae*. MAPK signal pathway is conserved in between appressorium formation and differentiation appressorium-like structures in *M. oryzae*. Pmk1 and Mps1 are essential for the development of the appressorium-like structures and penetration^40^. The Δ*pmk1* mutant could not form appressorium-like structures. In contrast, the Δ*mps1* mutant could form normal appressorium-like structures, but they failed to penetration and form invasive haphae^40^. In our studies, appressoria formed by germ tubes and appressorium-like structures developed by hyphal tips showed significant differences in the wild-type Guy11 and the mutants (Δ*Mocapn7*, Δ*Mocapn4*Δ*Mocapn7* and Δ*Mocapn9*Δ*Mocapn7*). Appressoria formed by the germ tubes showed similar shape and numbers, similar penetration of the barley epidermis, and similar colonization of the cells neighboring the penetration site in the wild-type Guy11 and the mutants (Δ*Mocapn7*, Δ*Mocapn4*Δ*Mocapn7* and Δ*Mocapn9*Δ*Mocapn7*). In contrast, appressorium-like structures developed by the hyphal tips of Δ*Mocapn7*, Δ*Mocapn4*Δ*Mocapn7* and Δ*Mocapn9*Δ*Mocapn7* showed delayed development and reduced penetration efficiency. It is conceivable that calpains might be essential for the development of the appressorium-like structures and penetration pegs.

In summary, calpains is a protein degradation system in *M. oryzae*. The targeted deletion of calpains affected on conidiation, sexual reproduction, cell wall integrity, and the pathogenicity of *M. oryzae* to rice and barley. Future experiments are needed to explore the interactions between calpains and other intracellular proteolytic systems, and the mechanism of the signal pathway regulating the calpains system in *M. oryzae*.

## Experimental procedures

### Fungal strains and growth conditions

The *M. oryzae* wild-type strain Guy11 and all the derivative transformants were routinely grown in complete medium (CM) at 25 °C with a 14 h light and 10 h dark cycle using fluorescent lights.

For growth analysis, the strains were grown on minimal medium (MM, 6 g NaNO_3_, 0.52 g KCl, 0.52 g MgSO_4_, 1.52 g KH_2_PO_4_, 10 g glucose, 0.5% biotin in 1 L distilled water, pH 6.5), OMA (oat meal agar, 30 g oats in 1 L distilled water) and 1/4 YG (1.3g yeast extract, 5 g glucose in 1 L distilled water) at 25 °C for 7 days under light-dark cycle conditions.

For DNA and RNA extractions, mycelia were harvested from 7-day-old cultures in liquid CM at 25 °C with shaking at 150-rpm. General molecular biology techniques for nucleic acid analysis were performed according to standard protocols.

### Construction of the knockout vectors and fungal transformation

An In-Fusion^®^ HD Cloning Kit was used to directionally clone the flanking fragments and the glufosinate ammonium-resistant gene (BAR) cassette fragment or the hygromycin-resistant gene (HPH) cassette fragment into the vector pKO1B (a generous gift from Dr. Jian-Ping Lu)^41^. The flanking fragments were amplified from genomic DNA of the wild-type strain Guy11 using Primer Star (TaKaRa, Japan) with the primers listed in Table S2. The BAR cassette fragment and the HPH cassette fragment were generated by PCR from pBARKS1 and pCB1003, respectively. The pKO1B vector was digested using the restriction enzymes *Hin*dIII and *Xba*I. All PCR products and linearized vectors were verified by agarose gel electrophoresis and then purified using a DNA Gel Extraction Kit (Axygen, China). The recombinant plasmids were transformed into competent cells of *E. coli* strain *DH5α*, and the sequences were confirmed by PCR with the primers listed in Table S2. Then the confirmed knockout plasmids were transformed into competent cells of *A. tumefaciens* strain AGL-1 using the freeze/thaw shock transformation method. Five strains of *A. tumefaciens* containing the confirmed knockout plasmids were obtained and designed MGG_06335-HPH-pKO1B, MGG_01072-HPH-pKO1B, MGG_07573-HPH-pKO1B, MGG_01072-BAR-pKO1B and MGG_04818-BAR-pKO1B. *Agrobacterium tumefaciens*-mediated transformation (ATMT) of *M. oryzae* was performed as previously described^42^. Transformants were selected on complete medium with 300 ug/ml glufosinate ammonium or 200 μg/ml hygromycin. Positive transformants were screened by GFP fluorescence screening and double PCR using the primers listed in Table S3. Only a single copy of the selectable marker gene (BAR/HPH/SUR) was identified in the null mutants by quantitative RT-PCR (single insertion of a knockout cassette). Southern blot analysis was performed according to the protocol provided with the digoxigenin (DIG) High Prime DNA Labeling and Detection Starter Kit I (Roche, Germany). Genomic DNA was digested by restriction enzymes (shown in Figure S1) and separated with a 0.7% agarose gel. The labeled probe was amplified from the genomic DNA of Guy11 using the primers listed in Table S4.

In the complementation assays, the full-length cDNAs of the calpain genes were amplified from the Guy11 cDNA library using the primers listed in Table S4. The amplified cDNA was cloned to the PKD1-GFP vector (a kind gift from Dr. Jian-Ping Lu) using the In-Fusion^®^ HD Cloning Kit (Clontech, USA) to generate the complementation vector. The confirmed complementation vector was transformed to generate the corresponding gene knockout mutant as described above.

### Quantitative RT-PCR

For quantitative RT-PCR (qPCR), total RNA was isolated using TRIzol following the manufacturer’s procedure (Molecular Research Center, USA). cDNA was reverse transcribed from 1 μg of total RNA with random 6-mers using a SYBR^®^ ExScript^TM^ RT-PCR Kit (TaKaRa, Japan) and was diluted 1:5 with nuclease-free water. Real-time PCR reactions were performed in a 20 μl volume containing 200 nM of each primer, 0.5 μl of cDNA and 10 μl of 2 × SYBR^®^ Premix Ex Taq (TaKaRa, Japan). Real-time PCR was performed on a Mastercycler^®^ ep Realplex (Eppendorf, USA). To analyze the relative abundance of the transcripts using the 2^−ΔΔCt^ method^43^, the average threshold cycle (Ct) was normalized to *β-tubulin* for each condition as 2^−ΔCt^. Fold changes were calculated as 2^−ΔΔCt^. The primer pairs used for qRT-PCR analyses are listed in Supplemental Table S5. The PCR reactions were repeated at least twice independently with three replicates, and a representative set of data are presented.

### Assays of phenotypic analysis

Phenotypic analyses, including formation of conidia, conidia germination and appressorium formation, were carried out as reported previously.

For conidia production, mycelia were grown on CM at 25 °C with a 12 h photophase for 7 days in 6-cm plates. The total conidia of a plate were harvested and counted using a hemocytometer. Conidial germination and appressorium formation were measured on plastic cover slips. The conidia at a suspension of 1 × 10^5^ conidia/ml in sterile distilled water were incubated at 25 °C for 2–48 h. The frequency of conidial germination and appressorium formation was determined at different time intervals. Each test was repeated three times with five replicates each time.

For fertility assays, Guy11 and mutants (Mat1-2) crossed with strain 2539 (Mat1-1) were cultured on OMA medium at 25 °C for the first 3 days with a 12-h photophase and were then placed under continuous white light at 22 °C for 4 weeks. The border between the mated individuals was examined for the capacity to form perithecia. The asci were collected and processed using the electron microscopy as described previously^30^. The semithin sections were examined under an Eclipse 80i microscope (Nikon).

The leaf infection assays were performed on rice (*Oryza sativa* cv. CO-39) and barley (*Hordeum vulgare* cv. ZJ-8) as previously described^30^. In cut leaf assays, 5 mm^2^ mycelia plugs and different concentrations of conidia were removed from 10-day-old CM media and deposited onto the upper surface of the isolated barley or rice leaves incubated at 25 °C with a 12-h photophase for 4 or 5 days. For spray infection assays, 14-day-old rice seedlings were used. The conidial suspension (1 × 10^4^ conidia/ml) was sprayed onto rice. Inoculated rice were placed in a dew chamber at 25 °C for 48 h in the dark and were then transferred to the growth chamber with a 12 h photophase for 5 days. Appressorium-like structure (ALS) development on barley leaves was examined by incubating 5 mm^2^ mycelia plugs on the leaves in a humid chamber for 48 h, 72 h, 96 h, and 120 h at 25 °C. Barley leaves were then excised, decolored with methanol and fixed with alcoholic lactophenol (95% alcohol: lactophenol = 2 : 1) at room temperature. The excised leaves were examined under an Eclipse 80i microscope (Nikon).

### Cell wall integrity assays

To test the effects of the cell wall-perturbing agents on Guy11 and the mutants, comparisons of the growth phenotypes of the wild-type strain Guy11 and the null mutants were performed on CM supplemented with various agents [200 μg/ml Congo red, 0.01% SDS and 100 μg/ml CFW (calcofluor white)]. The plates were incubated at 28 °C for 6 days in dark. The growth-inhibition rate was calculated as follows: growth inhibition rate = [(Diameter on CM−Diameter on CM with stress)/Diameter on CM] × 100. Each test was repeated three times, with three replicates each time.

To test the sensitivity of Guy11 and the mutants to the cell wall digesting enzyme, cultures were incubated in CM liquid medium at 25 °C and 150 rpm for 48 h, and 0.5 g samples of mycelia were harvested by filtration and digested in 2 ml of 0.7 M NaCl buffer with 7.5 mg/ml Glucanex (Sigma, USA). The protoplasts release and cell wall degradation were examined after a 30 °C incubation on a shaker at 80 rpm for 30 min.

## Additional Information

**How to cite this article**: Liu, X.-H. *et al*. Calpains are involved in asexual and sexual development, cell wall integrity and pathogenicity of the rice blast fungus. *Sci. Rep.* **6**, 31204; doi: 10.1038/srep31204 (2016).

## Supplementary Material

Supplementary Information
