# Corrigendum: Calpains are involved in asexual and sexual development, cell wall integrity and pathogenicity of the rice blast fungus

**DOI:** 10.1038/srep42618

**Published:** 2017-02-27

**Authors:** Xiao-Hong Liu, Guo-Ao Ning, Lu-Yao Huang, Ya-Hui Zhao, Bo Dong, Jian-Ping Lu, Fu-Cheng Lin

Scientific Reports 6: Article number: 31204; 10.1038/srep31204 published online: 08 09 2016; updated: 02 27 2017.

This Article contains typographical errors in the legend of Figure 4.

‘(**B**) The semithin sections of perithecia. 1, Δ*Mocapn14*; 2, Δ*Mocapn7*; 3, Δ*Mocapn9*; 4, Δ*Mocapn4*; 5, Δ*Mocapn7*; 6, Δ*Mocapn4*Δ*Mocapn7*; 7, Δ*Mocapn9*Δ*Mocapn7*’.

should read:

‘(**B**) The semithin sections of perithecia. 1, Guy11; 2, Δ*Mocapn*14; 3, Δ*Mocapn*9; 4, Δ*Mocapn4*; 5, Δ*Mocapn7*; 6, Δ*Mocapn4*Δ*Mocapn7*; 7, Δ*Mocapn9*Δ*Mocapn7*’.

