# Supplementary material for: Calpains are involved in asexual and sexual development, cell wall integrity and pathogenicity of the rice blast fungus
Source: Sci Rep. 2016 Aug 9;6:31204. doi: 10.1038/srep31204 (PMC4977516; doi:10.1038/srep31204)
Supplement: Supplementary Information [file srep31204-s1.pdf]

# Calpains are involved in asexual and sexual development, cell wall integrity and pathogenicity of the rice blast fungus

Xiao-Hong Liu, Guo-Ao Ning, Lu-Yao Huang, Ya-Hui Zhao, Bo Dong, Jian-Ping Lu, Fu-Cheng Lin

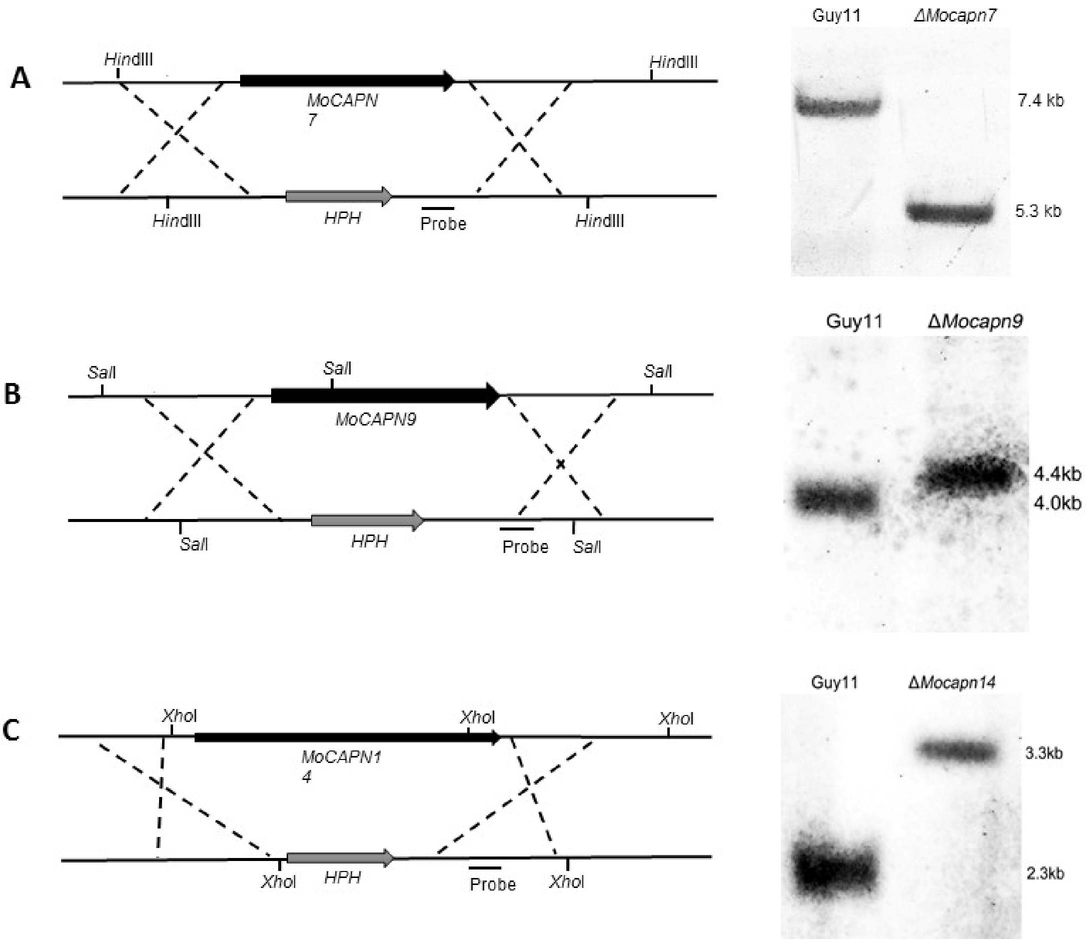


Figure S1 Targeted gene replacement of *MoCAPN7*, *MoCAPN9* and *MoCAPN14* and Southern blot analysis of the mutants. The gene deletion vectors were constructed using pKO1B as described in the Experimental procedures. The mutants were verified by Southern blot analysis. Restriction enzymes and probes for the Southern blot are labeled in the diagram of the gene replacement vectors. (A) *Hin*dIII-digested genomic DNA was hybridized with the 0.5 kb probe amplified with primers MGG_06335probe-F/R. A single band was shifted from 7.4 kb in Guy11 to 5.3 kb in Δ*Mocapn7*. A similar strategy was performed to verify deletion of *MoCAPN9* (B) and *MoCAPN14* (C).

**Table S1 Identification of calpains in *M. oryzae*.**

| Calpains in *Homo sapiens* | Homologues of calpains in *M. oryzae* | Designated Name in *M. oryzae* | Predicted Number of Amino Acids/Mass[a] | Sequence Identity/Similarity With Human Calpains (%) | Domains (Start-End)[b] | E-value[b] | Reference |
|---|---|---|---|---|---|---|---|
| Calpain1 | MGG_15810 | MoCapn1 | 1223/135.14 | 34/47 | Cyspc (165-502) | 8.98e-48 | Khan *et al*., (2014) |
| | | | | | PepC2 (173-292) | 3.9e-20 | |
| | | | | | PepC2 (310-490) | 5.7e-47 | |
| Calpain2 | MGG_08526 | MoCapn2 | 575/65.16 | 23/41 | Cyspc (205-574) | 1e-165 | this study[c] |
| | | | | | PepC2 (363-574) | 2.4e-13 | |
| Calpain3 | MGG_14872 | MoCapn3 | 798/90.4 | 30/42 | Cyspc (98-148) | 9.72e-44 | Khan *et al*., (2014) |
| | | | | | PepC2 (98-407) | 7.9e-69 | |
| Calpain4 | MGG_04818 | MoCapn4 | 257/29.52 | 33/53 | EFh (91-119) | 2e-10 | Khan *et al*., (2014) |
| | | | | | EFh (128-156) | 6e-12 | |
| | | | | | EFh (158-186) | 4e-11 | |
| | | | | | EFh (194-222) | 1e-9 | |
| | | | | | EFh (224-251) | 7e-10 | |
| Calpain7 | MGG_06335 | MoCapn7/PalB | 899/98.21 | 28/43 | Cyspc (98-443) | 1.89e-55 | this study |
| | | | | | CalpainIII (594-727) | 3.2e-9 | |
| | | | | | PepC2 (115-443) | 1.7e-32 | |
| Calpain9 | MGG_01072 | MoCapn9 | 678/73.26 | 34/59 | EFh (24-52) | 3e-11 | this study |
| | | | | | EFh (62-90) | 1e-10 | |
| | | | | | EFh (93-121) | 4e-12 | |
| | | | | | EFh (129-157) | 1e-13 | |

| | | | | | | | |
|---|---|---|---|---|---|---|---|
| | | | | | Mito_carr (357-468) | 7.8e-20 | |
| | | | | | Mito_carr (475-571) | 5.9e-22 | |
| | | | | | Mito_carr (583-677) | 2.4e-26 | |
| Calpain14 | MGG_07573 | MoCapn14 | 1060/118.39 | 32/44 | Cyspc (116-482) | 1.98e-26 | this study |
| | | | | | PepC2 (159-472) | 1.3e-58 | |
| Calpain1 | MGG_16201 | MoCapn1B | 150/17 | 27/52 | EFh (12-40) | 5.96-e | this study[c] |
| | | | | | EFh (48-76) | 1.19-e | |
| Calpain3 | | | | 26/46 | EFh (85-113) | 1.37-e | |
| | | | | | EFh (121-149) | 6.39-e | |

a. Mass is in kilo-daltons (KD).

b. The domains and the E-value was predicted using simple modular architecture research tool (SMART).

c. The null mutant was not got in this study.

**Table S2 List of primers for the flank fragments of the knockout vectors**

| Name | Sequence(5'-3') |
|---|---|
| MGG_06335upF | GCTGTACAAGTAAGAGCTCGGTACCCGGGGATCGAGCGATGGAGACATGATTAA |
| MGG_06335upR | ACCAAAATAGGCATTCATTGTTGACCTCCACTAAAAGCACCCAAAGAAGAGTAG |
| MGG_06335downF | CACTCGTCCGAGGGCAAAGGAATAGAGTAGATGTTCTGTTGAGATGGCTTGTTT |
| MGG_06335downR | TTAAGTTGGGTAACGCCAGGGTTTTCCCAGTCAGGCATGGGCATGGTAATAGTC |
| MGG_07573upF | GCTGTACAAGTAAGAGCTCGGTACCCGGGGATCTAAGACGCAGCGGGCTTGGAG |
| MGG_07573upR | ACCAAAATAGGCATTCATTGTTGACCTCCACTAATGGGCGGGAGAAATTAGGAA |
| MGG_07573downF | CACTCGTCCGAGGGCAAAGGAATAGAGTAGATGAAGATCGCTCGTTAAGACCCG |
| MGG07573downR | TTAAGTTGGGTAACGCCAGGGTTTTCCCAGTCATGACACTGTTTGCATCCTCGT |
| MGG_01072up1F | GCTGTACAAGTAAGAGCTCGGTACCCGGGGATCACAAGGGTTGGGTTCGTG |
| MGG_01072up1R | CCAAAAAGTGCTCCTTCAATATCATCTTCTCGGATTACTGCGGGTTCT |
| MGG_01072down1F | CCGTCCGGTCCTGCCCGTCACCGAGATTTAGAGTTATACGGCTACTATGACCT |
| MGG_01072down1R | TTAAGTTGGGTAACGCCAGGGTTTTCCCAGTCAAGTGCCACAGAAGCAATAC |
| MGG_04818upF | GCTGTACAAGTAAGAGCTCGGTACCCGGGGATCTTAGCATACCCATAACCAAA |
| MGG_04818upR | CCAAAAAGTGCTCCTTCAATATCATCTTCTAATAAGAAGTAGAAACCGAGAC |
| MGG_04818downF | CCGTCCGGTCCTGCCCGTCACCGAGATTTAGTGGCGACCATTTCAAGAC |
| MGG_04818downR | TTAAGTTGGGTAACGCCAGGGTTTTCCCAGTCAGCTCGGTGGCAACATCCT |
| MGG_01072upF | GCTGTACAAGTAAGAGCTCGGTACCCGGGGATCCCAGGACGTGGTCGTCATTAG |
| MGG_01072upR | ACCAAAATAGGCATTCATTGTTGACCTCCACTAAGTTGTCGCAGGTAGGTAGGC |

| | |
|---|---|
| MGG_01072downF | CACTCGTCCGAGGGCAAAGGAATAGAGTAGATGGGCTCGTTCTCCCATTGTAGT |
| MGG_01072downR | TTAAGTTGGGTAACGCCAGGGTTTTCCCAGTCACCTCGACGCACCGCCAAAGTA |
| MGG_16201upF | GCTGTACAAGTAAGAGCTCGGTACCCGGGGATCGCGGGAGAAGTAGTGGAGTTT |
| MGG_16201upR | ACCAAAATAGGCATTCATTGTTGACCTCCACTATTCGTCTTGTTGGGATGGTTG |
| MGG_16201downF | CACTCGTCCGAGGGCAAAGGAATAGAGTAGATGGGCGGTTCTTTGATGCCTTTA |
| MGG_16201downR | TTAAGTTGGGTAACGCCAGGGTTTTCCCAGTCATACCTTTCTGGGCGTTTCTGC |
| MGG_08526upF | GGTACCCGGGGATCCTCTAGAGTGTTCCTTTGCCCAGTG |
| MGG_08526upR | CTCCTTCAATATCATCTTCTCTTTCGTCTTTGCCTTCT |
| MGG_08526downF | TGCCCGTCACCGAGATTTAGGTTGATGGATGGTATTTGGGTA |
| MGG_08526downR | ACGACGGCCAGTGCCAAGCTTGGGTTTGTTCTGGGCTGAC |
| HPHF | TAGTGGAGGTCAACAATGAAT |
| HPHR | CATCTACTCTATTCCTTTGCC |
| BARKOF | AGAAGATGATATTGAAGGAGCA |
| BARKOR | CTAAATCTCGGTGACGGGCA |

**Table S3 List of primers for identification of the gene-deleted mutants**

| Name | Sequence(5’-3’) |
|---|---|
| MGG_06335up-hphF | AGAGCGTAGCATTGACCTTGA |
| MGG_06335up-hphR | ACGGTGGATGAACCATTGACT |
| MGG_06335innerF | CTGGGCAGTTAGCATCGT |
| MGG_06335innerR | TGGAGCAGCAACATAGGC |
| MGG_07573up-hphF | ATGTTATTGTTGTCGTCGTTG |
| MGG_07573up-hphR | CTACTCTATTCCTTTGCCCTC |
| MGG_07573innerF | TCGCACGCAAACTACCTC |
| MGG_07573innerR | CGTCCGTCTTCGCATCTT |
| MGG_01072inner1F | AGGAATGGCGGTAGGTTG |
| MGG_01072inner1R | GTTTGGCATTTGTGAGGC |
| MGG_01072up-barF | ATACAGCAGTGGCAAGTCG |
| MGG_01072uP-barR | TGAATCTGAAATAAAGGGAGG |
| MGG_04818innerF | CGAGCCCGAATCAGTAAG |
| MGG_04818innerR | GAAACCAATGGTGCCAGA |
| MGG_04818up-barF | AAGGTGGTTGGTTAGTGGT |
| MGG_04818up-barR | ATCTGAAATAAAGGGAGGAA |
| MGG_01072up-hphF | AGGCAGGACACGCTCTATTCA |
| MGG_01072up-hphR | GACAAACGCACCAAGTTATCG |
| MGG_01072innerF | AGGAATGGCGGTAGGTTG |
| MGG_01072inner | GTTTGGCATTTGTGAGGC |
| MGG_16201up-hphF | TTGACGGTTCGGTAAAGTTGT |
| MGG_16201up-hphR | CAGTTCGGTTTCAGGCAGGTC |
| MGG_16201innerF | ATGGTTCCCACAGGCTGATTC |
| MGG_16201innerR | TCGTTGTCTACCGCACCTACT |
| MGG_08526up-barF | GCAGCGATGCTTTACCCG |
| MGG_08526up_barR | AGCCCGATGACAGCGACCAC |
| MGG_08526innerF | GCAAGGCGGAGCGGTTTA |
| MGG_08526innerR | GAAGCGGTTGTAGGCGGTA |

**Table S4 List of primers for complementation and the probes of Southern blot**

| Name | Sequence(5’-3’) |
|---|---|
| MGG_06335comF | CACAATGGCCGGATCCATGGAAGCAAAATCATTGG |
| MGG_06335comR | TGCTCACCATCCCGGGGTCATCGTTGTACTCCCACTG |
| MGG_07573comF | CACAATGGCCGGATCCATGTTTGCACCAAGGGGCAAGG |
| MGG_07573comR | TGCTCACCATCCCGGGTGATTTGTCCTCTCCGTCC |
| MGG_01072comF | CACAATGGCCGGATCCATGAAGATCACAGATGTGGTC |
| MGG_01072comR | TGCTCACCATCCCGGGCTGTAGATTGAGGAACTTC |
| MGG_04818comF | CACAATGGCCGGATCCATGGCGTACAACCGACCCTAC |
| MGG_04818comR | TGCTCACCATCCCGGGCTTGAGCTGCTTCAGAATCTC |
| MGG_06335probeF | CTGACCAAGCACCAAGCG |
| MGG_06335probeR | CCCTGCGGGTATCCTATC |
| MGG_07573probeF | ATCAGGGCACAGATTCCA |
| MGG_07573probeR | ATCGGGCAAACTCTACAA |
| MGG_01072probeF | TGACAAGTGTATCAAGCGAAGT |
| MGG_01072probeR | GTGCCAGTGCCACAGAAG |

**Table S5 List of primers for quantitative RT-PCR**

| Name | Sequence(5’-3’) |
|---|---|
| MGG_06335RTF | CTGTTATGGATGTGCGAGTAGAG |
| MGG_06335RTR | TGCTGGAGAGTTTGGTGTC |
| MGG_07573RTF | TCAAACTTCGTAACCCCTGG |
| MGG_07573RTR | ACGTGATCCAAAAGACCGAG |
| MGG_01072RTF | AGAAAACCAAGCCCTCCAAG |
| MGG_01072RTR | GTTCTGGCTTTTGTGTTGACG |
| MGG_04818RTF | AACCAGAAACTTCCGTCTCC |
| MGG_04818RTR | TTCTCATATCGGCTTTGTGGG |
| MGG_14872RTF | CCGCTTTTGATTTTCGTGAGG |
| MGG_14872RTR | CATTAGAGGGAGCCAGGTTTC |
| MGG_15810RTF | GCCAAGGGAGACAAAACAATG |
| MGG_15810RTR | TTGTAGCCATACTCGGTTGC |
| MGG_08526RTF | CGGTTTACGCAAAAGGACATG |
| MGG_08526RTR | CCACCTTGACCTTGATGTTG |
| MGG_16201RTF | GAGCAAGTTTCCGAGTTCAAG |
| MGG_16201RTR | CGACCTCGTTGATCATATCCTG |
| HPHRTF | CGATAACTTGGTGCGTTTGTC |
| HPHRTR | TTGGATGCTTGGGTAGAATAGG |

| | |
|---|---|
| BARRTF | CGATAACTTGGTGCGTTTGTC |
| BARRTF | GGCTCATGGTTACTTCCTAATCG |
| β-tubulinRTF | ACAACTTCGTCTTCGGTCAG |
| β-tubulinRTF | GTGATCTGGAAACCCTGGAG |

## Table S6 Growth-inhibiting rate (%)*

| **Growth media** | **Guy11** | **Δ*Mocapn4*** | **Δ*Mocapn9*** | **Δ*Mocapn14*** | **Δ*Mocapn7*** | **Δ*Mocapn4*Δ*Mocapn7*** | **Δ*Mocapn9*Δ*Mocapn7*** |
|---|---|---|---|---|---|---|---|
| CM | 0 | 0 | 0 | 0 | 0 | 0 | 0 |
| CM+200μg/ml Congo red | 70.45±7.04$^{C}$** | 80.75±1.90$^{B}$ | 93.86±2.14$^{A}$ | 92.86±1.17$^{A}$ | 80.75±0.74$^{B}$ | 72.67±0.69$^{C}$ | 84.99±0.26$^{B}$ |
| CM+100μg/ml CFW | 71.57±2.97$^{C}$ | 82.65±0.61$^{B}$ | 85.97±2.21$^{A}$ | 87.60±2.52$^{A}$ | 82.41±1.51$^{B}$ | 73.05±1.03$^{C}$ | 88.74±1.17$^{A}$ |
| CM+0.01%SDS | 44.29±0.85$^{B}$ | 44.34±0.60$^{B}$ | 43.61±1.11$^{C}$ | 44.03±1.25$^{B}$ | 47.07±2.05$^{A}$ | 43.58±0.80$^{C}$ | 47.73±0.89$^{A}$ |

*Growth-inhibition rate of strains on CM plates supplemented with 100μg/ml CFW, 0.01%SDS and 200μg/ml Congo red at 28 °C in dark for 6 days. Growth-inhibition rates were calculated as described in Experimental procedures.

** Different letters indicate a statistically significant difference ($P<0.01$).
